# Assessment of the Therapeutic Potential of Metallothionein-II Application in Focal Cerebral Ischemia *In Vitro* and *In Vivo*

**DOI:** 10.1371/journal.pone.0144035

**Published:** 2015-12-14

**Authors:** Abass Eidizadeh, Manuel Khajehalichalehshtari, Dorette Freyer, George Trendelenburg

**Affiliations:** 1 Dept. of Neurology, University Medical Center Göttingen, Robert-Koch Str. 40, 37075, Göttingen, Germany; 2 Dept. of Neurology, Charité University Medical Center, Charitéplatz 1, 10117, Berlin, Germany; School of Pharmacy, Texas Tech University HSC, UNITED STATES

## Abstract

Metallothionein-II (MT-II) is an ubiquitously expressed small-molecular-weight protein and highly induced in various species and tissues upon stress, inflammation, and ischemia. MT-deficiency exacerbates ischemic injury in rodent stroke models *in vitro* and *in vivo*. However, there is conflicting data on the potential neuroprotective effect of exogenously applied metallothionein. Thus, we applied MT-II in an *in vitro* stroke model and intraperitoneally (*i*.*p*.) in two *in vivo* standard models of transient middle cerebral artery occlusion (MCAO) (a ‘stringent’ one [60min MCAO/48h reperfusion] and a ‘mild’ one [30min MCAO/72h reperfusion]), as well as *i*.*v*. together with recombinant tissue plasminogen activator (rtPA) to evaluate if exogenous MT-II-application protects against ischemic stroke. Whereas MT-II did not protect against 60min MCAO, there was a significant reduction of direct and indirect infarct volumes and neurological deficit in the MT-II (*i*.*p*.) treated animals in the ‘mild’ model at 3d after MCAO. Furthermore, MT-II also improved survival of the mice after MCAO, suppressed TNF-α mRNA induction in ischemic brain tissue, and protected primary neuronal cells against oxygen-glucose-deprivation *in vitro*. Thus, exogenous application of MT-II protects against ischemic injury *in vitro* and *in vivo*. However, long-term studies with different species and larger sampling sizes are required before a clinical use can be envisaged.

## Introduction

Ischemic stroke is the second leading cause of death worldwide and the absolute number of stroke patients is still increasing despite a decrease in age-standardized rates of stroke mortality in the last two decades [1]. Ischemic brain injury is caused by a complex cascade of pathophysiological events including exitotoxicity, peri-infarct depolarizations, apoptosis, and inflammation [2,3]. Manifold interventions have been proven successful in experimental stroke studies. However, most of them failed in the clinical studies with human stroke patients [4].

Metallothioneins (MT) are small molecular weight (6–7 kDa) cytoplasmic metal-binding proteins with a high content of cysteine residues. Four family members of the MTs are expressed in mice and have been involved in various signaling pathways, including protection against exitotoxicity, inflammation, axonal sprouting, or regulation of apoptosis [5]. MT-I and MT-II are thought to act similarly and their neuroprotective effect has been ascribed to their abilities to scavenge toxic divalent metal-ions like zinc, copper, and cadmium, as well as protect against reactive oxygen species (ROS) with its cystein-rich domains [6–8]. There is also speculation that MTs regulate Zn-containing transcription factors due to their zinc-finger domains and their metal-binding capabilities [9].

In contrast to MT-III, which in the central nervous system (CNS) is mainly expressed in neurons, MT-II is predominantly expressed in astroglia [10] and is induced by stress, inflammation, and metals [11]. MT-II is also secreted in the extracellular space during conditions of tissue stress or damage and where it is thought to modulate immune responses, and the resolution of tissue damage [12]. Extracellular MT-II was postulated to mediate its effects on neuronal cells through membrane receptors from the low-density lipoprotein family, mostly lipoprotein receptor-1 and -2 (megalin) [5,13]. MT was shown to regulate the expression of growth and trophic hormones responsible for angiogenesis, neurogenesis, axonal sprouting, as well as anti-inflammatory cytokines. Thus, it promotes regeneration, repair, and neuroprotective effects after brain-injury [14,15]. MTs reveal neuroprotective and anti-inflammatory abilities in murine models of diseases of the central nervous system (CNS), such as multiple sclerosis and Parkinson’s disease [5,16,17]. The contribution of MT-I and–II in traumatic brain injury was demonstrated by the use of MT-I/II-double-knockout mice, which had a severely depressed wound healing when compared to wild-type mice [18]. Moreover, a whole transcriptome screening approach identified MT-II as the most significantly induced gene 12 hours after the start of reperfusion in a murine standard model of focal cerebral ischemia [19]. The neuroprotective potential of MT in experimental stroke was underlined by the reduced infarct volumes in mice over-expressing MT-I [20], as well as by the significantly enlarged infarct volumes in MT-I/-II double knockout mice after transient focal cerebral ischemia [19]. Moreover, metallothionein-III (a brain specific MT isoform) is induced after stroke in neurons and astrocytes. MT-III knockout mice had aggravated ischemic brain damage after transient focal ischemia [21], but not after permanent cerebral ischemia [22]. MT-III displays strong ROS scavenging properties but impairs neurite outgrowth and neuronal survival of neurons *in vitro* [23]. A PEP-1-MT-III fusion protein protected against oxidative stress induced cell death *in vitro* and *in vivo* [24]. Various *in vitro* and *in vivo* studies since then support this robust neuroprotective effect in different models of injury, inflammation, or neurodegeneration [25]. For example, intraperitoneal treatment with MT-I/II in a model of focal cryolesion brain injury reduced oxidative stress and neuronal apoptosis in a vehicle-controlled study [26]. The exact mechanism of neuroprotective abilities of extracellular metallothionein remains unclear, but potentially involves lipoprotein-receptor related proteins (LRP), such as LRP2 or megalin [25].

Interestingly, despite the robust neuroprotective effects in various models of CNS injury, the high degree of expression of metallothionein mRNA in the central nervous system [19], as well as its significant induction under circumstances of stress and danger, there is almost no data on exogenously applied metallothionein in stroke up until now. Only one very recent study, which was published after the start of our project, reported on a protective effect of MT-application *i*.*p*. in a rat model of transient stroke [27]. It was further reported that isofluorane preconditioning protects neuronal and glial cells against oxygen-glucose deprivation (OGD) *in vitro* and is mediated by MT [28]. However, there is also discussion on whether exogenous MT can reach the CNS if applied *i*.*m*. or *i*.*p*. [29]. Thus, interpretation of the significance of the therapeutic potential of MT in stroke is currently hindered by the limited data (e.g. with regard to different species, stroke models, or ways of application).

In accordance with the recommendations of the STAIR criteria [30], we therefore evaluated the MT-effect in different experimental stroke models *in vitro* and *in vivo*: MT-II was applied *in vivo* by different routes (*i*.*p*. vs *i*.*v*. and with vs. without co-administration of rtPA, respectively) in combination with middle cerebral artery occlusion (MCAO) models in mice, as well as *in vitro* in combination with oxygen-glucose-deprivation (OGD). Our results revealed a significant benefit of MT-II treatment with regard to stroke sizes and neurological deficit in standard experimental stroke models *in vitro* and *in vivo*, but also with regard to animal survival after experimental stroke.

However, model-specific differences were observed and protective effect sizes did not reach those observed with MT-I/II deficient or MT-over-expressing transgenic mice [19,20]. This potentially explained by the different locations of action (extracellular vs intracellular way of action), or by the route of application [29].

## Material and Methods

### Primary neuronal cell cultures

MEM, Trypsin/EDTA, HEPES, Penicillin/Streptomycin, L-glutamine, FBS, Poly-L-Lysin, CollagenG were purchased from Biochrom GmbH (Berlin, Germany), Neurobasalmedium, B27, PBS were from Life Technologies (Darmstadt, Germany), Zn-metallothionein-II from Enzo Life Sciences (Lörrach, Germany), and all other chemicals from Sigma-Aldrich (München, Germany). Primary neuronal cell cultures were obtained as described previously [31]. Briefly, embryonic brains were obtained from fetal rats at E17, the cerebral cortex was dissected, incubated for 15 min in trypsin/EDTA (0.05/0.02% w/v in PBS w/o Ca^++^, Mg^++^) at 37°C, rinsed twice with PBS w/o Ca^++^, Mg^++^ and once with dissociation medium (MEM Eagle with 10% fetal bovine serum, 10 mM HEPES, 44mM glucose, 100 U penicillin/Streptomycin/ml, 2mM L-glutamine, 100 I.E. insulin/l), dissociated by Pasteur pipette in dissociation medium. Cells were pelleted by centrifugation at 210 g for 2 min (room temperature), resuspended in starter medium (Neurobasal Medium and supplement B27, 100 U/ml Penicillin, 100μg/ml Streptomycin, 0.5 mM L-glutamine, 25 μM glutamate), and seeded in 24 well plates in a density of 150 000 cells/cm^2^. Plates were coated with Poly-L-Lysin (5μg/ml over night at 4°C), washed once with PBS, incubated with Collagenmedium (MEM Eagle with 10% FBS, 10 mM HEPES, 100 U penicillin/Streptomycin/ml, 0.004% CollagenG) for 1h at 37°C, 5% CO_2_, washed twice with PBS with Ca^++^, Mg^++^ and filled by starter medium. Cultures were kept at 36.5°C and 5% CO_2_. After 4 days in vitro, 200 μl medium/well were replaced with 300 μl cultivating medium (starter medium without glutamate). The OGD experiments took place on div 9.

### Preconditioning and Oxygen-Glucose deprivation (OGD)

Primary neuronal cell cultures were preconditioned for 12 h with MT-II (0, 0.1, 1, 5 μg/ml). For OGD, medium was removed and collect separately for every condition, cell cultures were washed twice with BSS_0_, followed by an incubation in 400 μl BSS_0_/well at 36.5°C, 5% CO_2_, 0.3% O_2_ for 3.5h (in-vivo400, Ruskinn). OGD controls were washed twice with BSS_20_ and were simultaneously kept with BSS_20_ (BSS_0_ + 20 mM glucose) in the cell culture incubator. After OGD, BSS solution was replaced by collected medium. BSS_0_: 116 mM NaCl, 5.4 mM KCl, 0.8 mM MgSO_4_, 1 mM NaH_2_PO_4_, 26.2 mM NaHCO_3_, 10 μM glycine, 1.8 mM CaCl_2_, 1 mM HEPES. 24h after OGD, the neuronal survival was analyzed by LDH release which was measured as described by Bruer et al. [32] and Koh & Choi. [33] LDH release was normalized to total LDH.

### Animals

8–11 weeks old male C57BL/6N mice (Charles River, Cologne, Germany) were housed in the local animal facility of the University Medical Center Göttingen under diurnal lighting conditions and allowed access to food and water *ad libitum*. Handling and surgery were performed according to the rules of the Guidelines for the Use of Animals in Neuroscience Research (Society of Neuroscience) and according to institutional and national guidelines. Experiments were approved by the local institutional veterinary and ethics commission (LaVeS / Niedersächsisches Landesamt für Verbraucherschutz und Lebensmittelsicherheit, TVA No. 12/0849). Mean body weights of mice used for experiments were (mean ± SD): 23.0 ± 0.7g (60min MCAO i.p.), 25.2 ± 0.8g (30min MCAO i.p.), and 25.7g ± 1.4g (*i*.*v*. treatment). Experiments were performed in a randomized manner and by investigators blinded to the groups of treatment.

### Middle cerebral artery occlusion (MCAO)

Transient focal cerebral ischemia (MCAO) was induced with a silicone-coated nylon-monofilament (Doccol Corporation, Sharon, USA), which was inserted into the common carotid artery and then pushed forward through the internal carotid artery into the middle cerebral artery as described previously [34]. 11mm filaments were used with a coating-zone of 9–10mm and a diameter of 0.21mm. The anesthesia was performed using 4% isoflurane for induction and 2% isoflurane, 70% N_2_O and 30% O_2_ for maintaining through a face mask (*i*.*p*. treated groups), or 1% isoflurane in combination with buprenorphine *i*.*p*. (0.1 mg/kg body weight) (Temgesic, Essex Pharma, Munich, Germany) (*i*.*v*. treated groups) to allow spontaneous breathing during anesthesia. The operation-time did not exceed 15min. Different standard models of MCAO were performed. One central purpose of our investigation was to evaluate the therapeutic potential of MT-II in two different standard models of transient focal cerebral ischemia. A stringent one (with 60min occlusion time and 48h reperfusion time) and a mild one (with only 30min occlusion time and 72h reperfusion time) were used as describe before [35]. Moreover, to evaluate potential detrimental interactions of exogenously *i*.*v*.-applied MT-II with, or without the clinical standard treatment of acute stroke (*i*.*v*.*-*thrombolysis using rtPA), we adopted the MCAO model used in the study of Trendelenburg et al. [19] which uses 45min MCAO in combination with 48h of reperfusion. The body temperature was continuously measured during surgery and kept between 37.0 and 37.5°C with a heating pad. After surgery, mice were kept in a recovery chamber for 2h. Twenty-four hours after reperfusion, buprenorphine i.p. (0.1 mg/kg body weight) (Temgesic, Essex Pharma, Munich, Germany) was applied to prevent pain. Efficiency of occlusion and reperfusion of the middle cerebral artery was monitored by laser doppler flowmetry (Perimed, Stockholm, Sweden) in a selected group of animals (n = 15) before starting with the main project.

### Mortality rates and exclusion/euthanasia criteria

Animals without a demarcated infarct and animals that died within 6 hours after MCAO were excluded from any analysis as death was assumed to be a direct complication of the surgical procedure. To ensure human endpoints during the study, specific euthanasia criteria were defined (see local ethic approval LaVeS / No.33.9-42502-04-12/849). According to these, animals that had lost 20% of their initial body weight within 48 hours or had measured surficial body temperatures lower than 24°C without recovery within 24 hours were deeply anaesthetized, cervically dislocated and finally decapitated. Mortality is summarized as a table in the supplement (S1 Table). Mortality rates: In the 60min MCAO group, 2 mice died peri-operatively in each group (together n = 4), and 1 mouse in the vehicle-treated control group died before the end of 48h reperfusion. In the 30min MCAO experiment, 1 of the MT-II treated mice died peri-operatively and 2 vehicle treated mice died postoperatively before 48h. In the *i*.*v*.*-*treatment experiment, 11 of the 60 animals died during operation or in the following 6h of reperfusion (peri-operative mortality). During the following reperfusion time, 5 out of 13 vehicle-treated mice, none of the 12 MT-II-treated mice, 2 out of 13 rtPA and vehicle treated mice, and none of the 11 MT-II and rtPA-treated mice died within 48h reperfusion. For qRT-PCR experiments, 1 out of 12 mice died peri-operatively, and one further mouse died within the 72h reperfusion (both in the MT-II treated group).

### Metallothionein (MT) treatment

Zn_7_-metallothionein-II isolated from rabbit liver (MT-II) was supplied from Enzo Life Sciences, (Cat.No.: ALX-202-071-C500, Lörrach, Germany) in cell culture grade purification (≥95%. ~7 Zn per molecule). It is a mixture of MT isoforms, mainly containing MT-2a and minor portions of MT-2b and MT-2c. MT-II is essentially free of cadmium and copper and replaced by zinc ions. Its formulation is solved liquid in 25mM TRIS/HCl, pH 8.0, 50mM NaCl. For injection, MT-II was diluted in 0.9% sterile NaCl. No endotoxin activity in mice is known.

**MT-II i.p.** 5μg / 10g body-weight MT-II was applied intraperitoneally (*i*.*p*.) every 12h, starting with the begin of the occlusion of the middle cerebral artery as described previously. [26] The control group was treated the same way using 0.9% NaCl as vehicle control instead of MT-II.

**MT-II i.v.** 5μg/10g body-weight MT-II was applied intravenously (*i*.*v*.*)* at 5min after start of reperfusion by the use of a small gage needle (Cannula Sterican 30 G 0.30 x 12 mm, B. Braun Melsungen AG, Melsungen, Germany) in the anterior facial vein under direct visual control [36]. rtPA (Actilyse, Boehringer Ingelheim, Germany) or 0.9% NaCl as vehicle control, respectively was co-administered *i*.*v*. with MT-II in specified groups 0.9mg/kg body-weight, as used in human patients [37].

### Infarct volumetry

For TTC-staining, mice were deeply anaesthetized and the brains were removed from the skull 48h after reperfusion. Brain tissues were cut into slices of 2 mm depth and stained with TTC (2,3,5–triphenyl-tetrazolium-chloride) (Merck, Darmstadt, Germany). For hematoxylin staining, mice were deeply anaesthetized and perfused through cardial injection with 75ml PBS and 75ml 4% paraformaldehyde (PFA). The brains were removed and incubated in 4% PFA for 24h. For preparation with the cryomicrotom, the brains were incubated in 30% sucrose-dilution for 48h, sectioned, and stained in hematoxylin (Merck, Darmstadt, Germany) for 10min. 14μm coronar cryosections at interaural positions 6.6 (No. V), 5.3 (No. IV), 3.9 (No. III), 1.9 (No. II), and 0mm (No. I) from Bregma were used. Digitized sections were used to determine the infarcted area of the ipsilateral brain hemisphere (direct infarct), the non-infarct area (ipsilateral), and the size of the contralateral (non-ischemic) hemisphere by the use of Image J software (NIH, USA) [38]. Infarct volumes (mm^3^) were calculated as described before [34,35]: indirect infarct volume was calculated by the difference of the size of the contralateral hemisphere minus the non-infarct volume of the ipsilateral hemisphere to correct for brain swelling (S1 Fig). Calculated brain swelling was defined as the difference between direct and indirect infarct volumes.

### Statistic analysis

Power calculation was performed using SISA Binominal [39]: based on the observed variances of previous experiments, the MCAO experiments were powered (α = 0.05; ß = 0.8) to detect effect sizes *d* [40] of at least 1, i.e. of one standard deviation. Figures and statistical analysis were performed using GraphPadPrism Software (Version 5, La Jolla, USA). Statistical analysis was performed using Mann-Whitney-U-test if not stated otherwise. If values were normally distributed, student’s one-side t-test was performed (e.g. for fold change calculation in gene expression analysis). Analysis of multiple normally distributed groups was performed by ANOVA. The log-rank test was used for the statistical analysis of animal survival (Kaplan-Meier plot). P-values below 0.05 were considered statistically significant. Values (if not stated otherwise) were given as mean ± standard error of the mean (SEM).

### Neurological Score

For clinical scoring of the neurological deficits, the neurological score was determined according to Bederson et al. [41] and as modified by Hara et al. [42]. Scoring was performed every 24h, starting directly before MCAO. A score of 0 represents no deficits, 1 represents an extension deficit in the contralateral leg, 2 a hemiparesis with circling, 3 loss of postural reflexes, and 4 death. Mice that died before the end of reperfusion time were excluded from infarct volumetry or assessment of cell numbers, but included in evaluation of neurological deficit (neuroscore 4).

### Immunohistofluorescence

Mice were euthanized at 48h after reperfusion. Brains were removed after transcardial perfusion, post-fixed in 4% PFA and 30% sucrose (each over night) and embedded for cryogenic cuttings. 12μm sections were air-dried and blocked at 4°C over night using a solution of 0,25% Triton-X-100 and 5% donkey serum in TBS followed by an incubation with mouse monoclonal anti-mouse NeuN-antibody (1:500, Merck Millipore, USA), rabbit polyclonal anti-mouse Iba1-antibody (1:1000, Wako Chemicals GmbH, Neuss) for 24h at 4°C. Incubation with secondary antibodies (Cy5-conjugated donkey anti-mouse, Cy3-conjugated donkey anti-rabbit, Jackson ImmunoResearch Europe Ltd., Suffol, UK), was performed for 60min at room temperature in the dark, and followed by counterstaining with DAPI (Merck Millipore, Darmstadt, Germany). Slides were scanned with AxioCamMRm and processed with Zen software Version 1.0.0.0 (both Zeiss, Jena, Germany).

### Histological quantification of neuronal and inflammatory cells

The immunofluorescence labeled sections were acquired with Zeiss Axio Examiner Microscope (Oberkochen, Germany). Inflammatory cells (macrophages, respectively microglia) were determined by counting all Iba1-positive cells at interaural position No.III (distance to bregma 3.9 mm) in the ischemic (ipsilateral) hemisphere.

### Relative Quantification of Gene Expression with Real-Time PCR

RNA was separately isolated from the ischemic (ipsilateral) and non-ischemic (contralateral) homogenized brain hemispheres, using TRIzol Reagent (Life Technologies, Carlsbad, CA, USA) according to manufacture protocol. Contaminating DNA was removed by DNase digest with DNase I (Sigma-Aldrich, St.Louis, MO, USA) using manufacture protocol. Concentration of RNA was photometrically determined with NanoDrop 2000c (Peqlab, Erlangen, Germany) and 1μg total RNA was used with reverse transcription system iScript cDNA Synthesis Kit (Bio-Rad Laboratories, Hercules, CA, USA) as recommended by the manufacturer. The cDNA was eluted in a final volume of 100μl RNA-free-Water in 1:20 concentration. Quantitative real-time PCR was performed with SYBR Green I Master-Mix (Roche, Mannheim, Germany) using the LightCycler 480 (Roche, Mannheim, Germany). The ΔΔCt method was used for the calculation of normalized relative gen-expressions. ΔCt was calculated by normalizing the Ct-values of target-gens to a reference-gen (hypoxanthine-guanine phosphoribosyl transferase 1 / HPRT-I). ΔΔCt was calculated, taking the difference between the ΔCt of ipsilateral hemisphere and the contralateral hemisphere. Final results were expressed as fold changes between the different treated groups. Primer sequences for HPRT-I: HPRT-I_*forward*_: GGT TAA GCA GTA CAG CCC CA; HPRT-I_*reverse*_: TGG CCA CAG GAC TAG AAC AC; Interleukin-1β: IL-1β_*forward*_: GGA AAG AAT CTA TAC CTG TCC TGT GTA ATG; IL-1β_*reverse*_: CAT TAG AAA CAG TCC AGC CCA TAC TTT AG; tumor necrosis factor α: TNFα_*forward*_: CCT CAC ACT CAG ATC ATC TTC TCA AAA TTC; TNFα_*reverse*_: CTT TCT CCT GGT ATG AGA TAG CAA ATC G; apoptosis-associated speck-like protein containing a CARD (ASC): ASC_*forward*_: AAC TGC GAG AAG GCT ATG GG; ASC_*reverse*_: GTC CAC TTC TGT GAC CCT GG; Caspase-1: Casp1_*forward*_: CCC AGA AGT TAT GGA AAG AAA ATC CTT CAG; Casp1_*reverse*_: GGA TAC CAT GAG ACA TGA ATA CAA GGA AAG.

## Results

### Extracellular metallothionein-II protects primary neuronal cells against oxygen-glucose deprivation (OGD)

**Fig 1:**
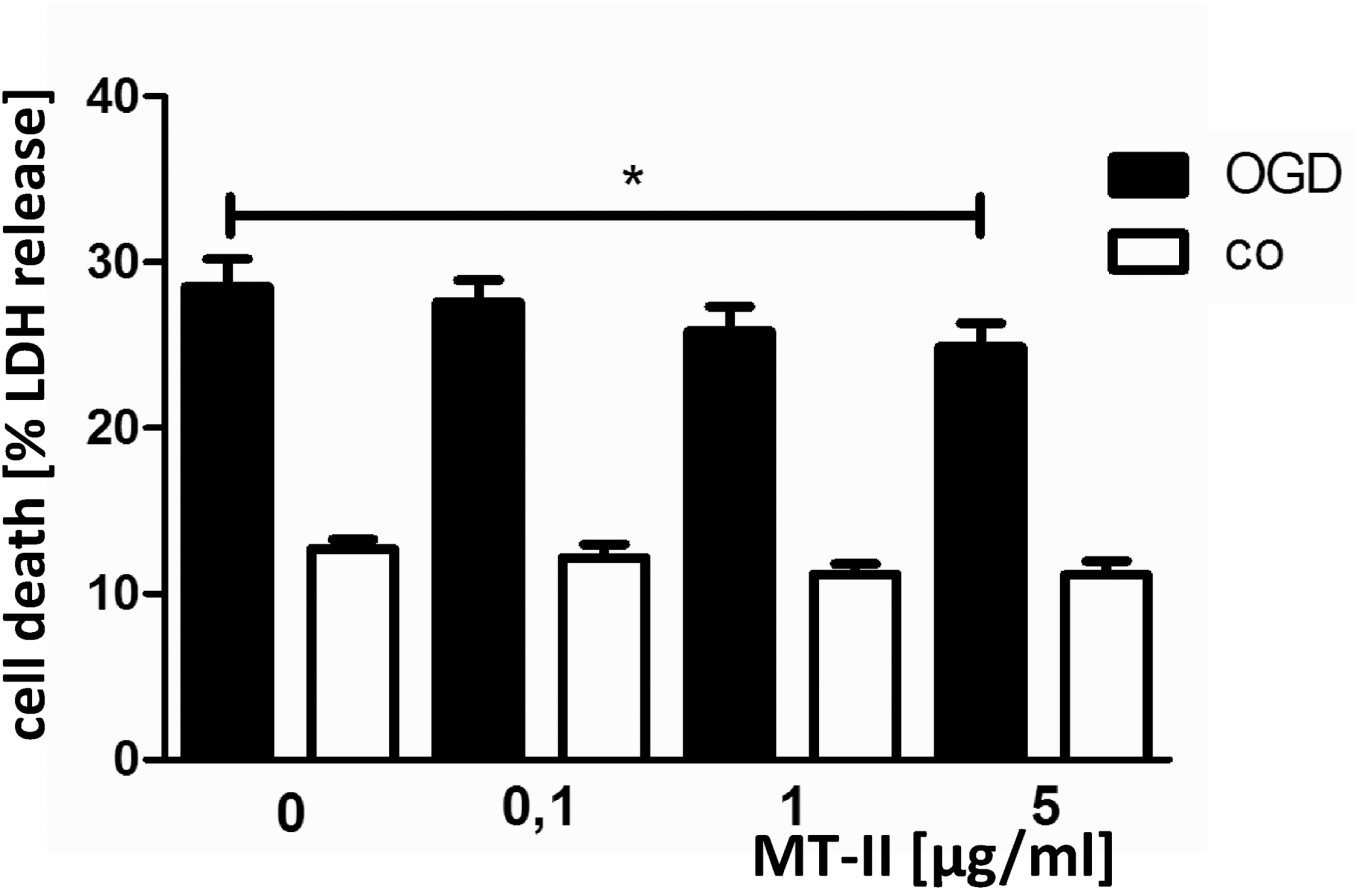
Primary neuronal cells treated with metallothionein-II (MT-II) are protected against oxygen glucose deprivation (OGD) *in vitro*. Shown is the relative LDH release of primary neuronal cells after OGD (shown as “cell death (%LDH release)”). Cells are treated with various different concentrations of MT-II before induction of oxygen and glucose deprivation (**MT-II**: primary neuronal cells pretreated with metallothionein-II 12 h before OGD; **co**: control cells without OGD [BSS_20_]). (*p = 0.0198, as calculated by Mann Whitney test; one-tailed).

First, we tested if exogenous metallothionein-II is able to protect primary neuronal cells against ischemic injury *in vitro*. For that purpose, we applied a standard *in vitro* model of stroke to rodent primary neuronal cultures. Rat primary neuronal cells incubated with various concentrations of exogenous metallothionein-II were exposed to oxygen- glucose deprivation (OGD) for 3.5h. As shown in Fig 1, metallothionein-II dose-dependently protected against OGD. Increasing the concentration of metallothionein-II up to 5μg/ml in primary neuronal cell culture provokes an increasing degree of protection against OGD and resulted in a significant reduction of neuronal cell death after OGD: relative neuronal cell death (related to total lactate dehydrogenase [LDH]) decreased from 28.5% (without MT-II) down to 24.9% (*p* = 0.0198) when cells were incubated with 5μg/ml MT-II (Fig 1). Thus, metallothionein-II dose-dependently protects against oxygen-glucose deprivation *in vitro*.

### Evaluation of the neuroprotective potential of MT-II in vivo by the use of MT-II application i.p. in two different standard MCAO models

**Fig 2:**
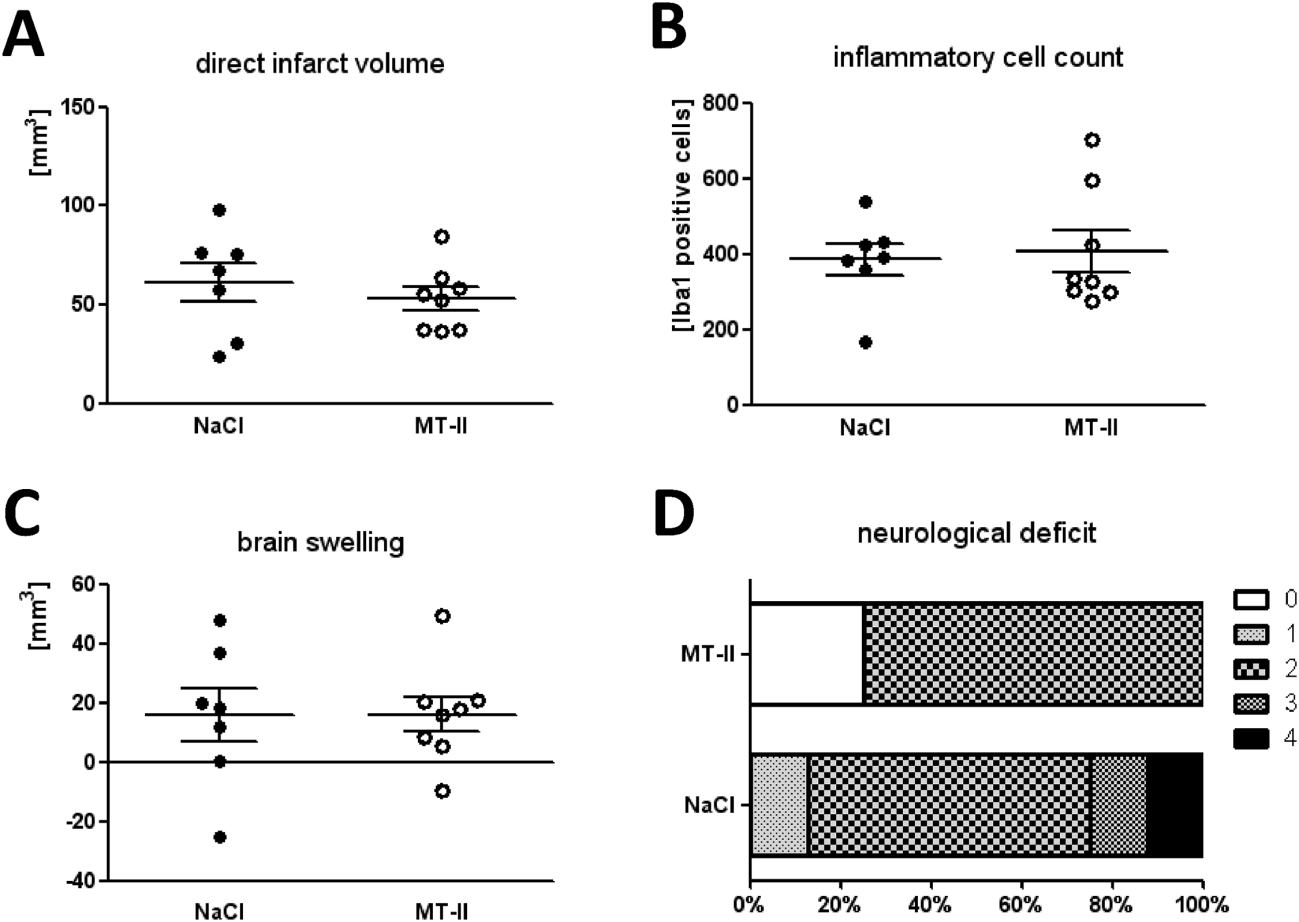
Effect of metallothionein-II *i*.*p*. treatment *in vivo* 48h after MCAO for 60 min. Infarct volumes of vehicle (0.9% NaCl) (n = 7) or MT-II (*i*.*p*.) treated (n = 8) male adult wildtype C57BL/6N mice after 60min MCAO and 48h reperfusion. **A**: direct infarct volumes. **B**: inflammatory cell count in the ischemic hemisphere as determined by counting Iba1-positive cells in whole ischemic brain hemispheres of mice at 48h of reperfusion after 60min MCAO. Inflammatory cell accumulation (macrophages and activated microglia) was determined by counting Iba1-positive cells at interaural position No.III (distance to bregma 3.9mm) in the whole ischemic/ipsilateral hemisphere. **C**: Brain swelling, as calculated by the difference between direct and indirect infarct volumes. Whereas indirect infarct volumes were calculated as the volume of the contralateral hemisphere minus the non-infarcted volume of the ipsilateral/ischemic hemisphere (p-values between groups were >0.05 as calculated by Mann Whitney U-Test, one-tailed). **D:** neurological deficit of mice at 48h after induction of MCAO as determined by a modified Bederson score [41]: 0 represents no deficits, 1 represents an extension deficit in the contralateral leg, 2 a hemiparesis with circling, 3 loss of postural reflexes, and 4 death.

To investigate the therapeutic potential of metallothionein(MT)-II-treatment in experimental stroke *in vivo*, we induced focal transient cerebral ischemia in wild-type mice via two different standard models: a more severe stroke model (60min occlusion time) and a mild one (30 min occlusion time) [35]. For that purpose, metallothionein-II was applied *i*.*p*. every 12h in male adult C57BL/6N wild-type mice starting with the begin of the occlusion (MCAO). MT-II treated mice were compared to vehicle (0.9% NaCl) treated mice of the same age and gender (Fig 2).

#### 60min MCAO/48h reperfusion

At 48h reperfusion after 60min MCAO (severe MCAO), there was no significant difference of infarct volumes between both treatments (MT-II vs. vehicle), despite a tendency towards reduced infarct volumes in metallothionein-II-treated animals: direct infarct volumes were 61 ± 10mm^3^ (*n* = 7) in the vehicle-group, and 53 ± 6mm^3^ (*n* = 8) in the MT-II group (n.s.) (Fig 2A). Indirect infarct volumes were 45.5 ± 4mm^3^ (control group), respectively 37 ± 5.5mm^3^ (MT-II group) (n.s.) (data not shown), and calculated brain swelling 15.8 ± 9mm^3^ (control group), respectively 16 ± 6mm^3^ (MT-II-group) (n.s.) (Fig 2C). Analysis of inflammatory cell accumulation by counting Iba1-positive cells in the whole ischemic hemisphere did not reveal a significant difference between both treatment groups (vehicle-group: 384 ± 42; MT-II treated group: 407 ± 56; n_*vehicle*_ = 7; n_*MT-II*_ = 8; *p* = n.s.) (Fig 2B). Moreover, there was no significant difference in the neurological deficit in both groups after induction of cerebral ischemia for 60min, neither at 24h (n.s.), nor at 48h (n.s.) of reperfusion (Fig 2D).

#### 30min MCAO/72h reperfusion

**Fig 3:**
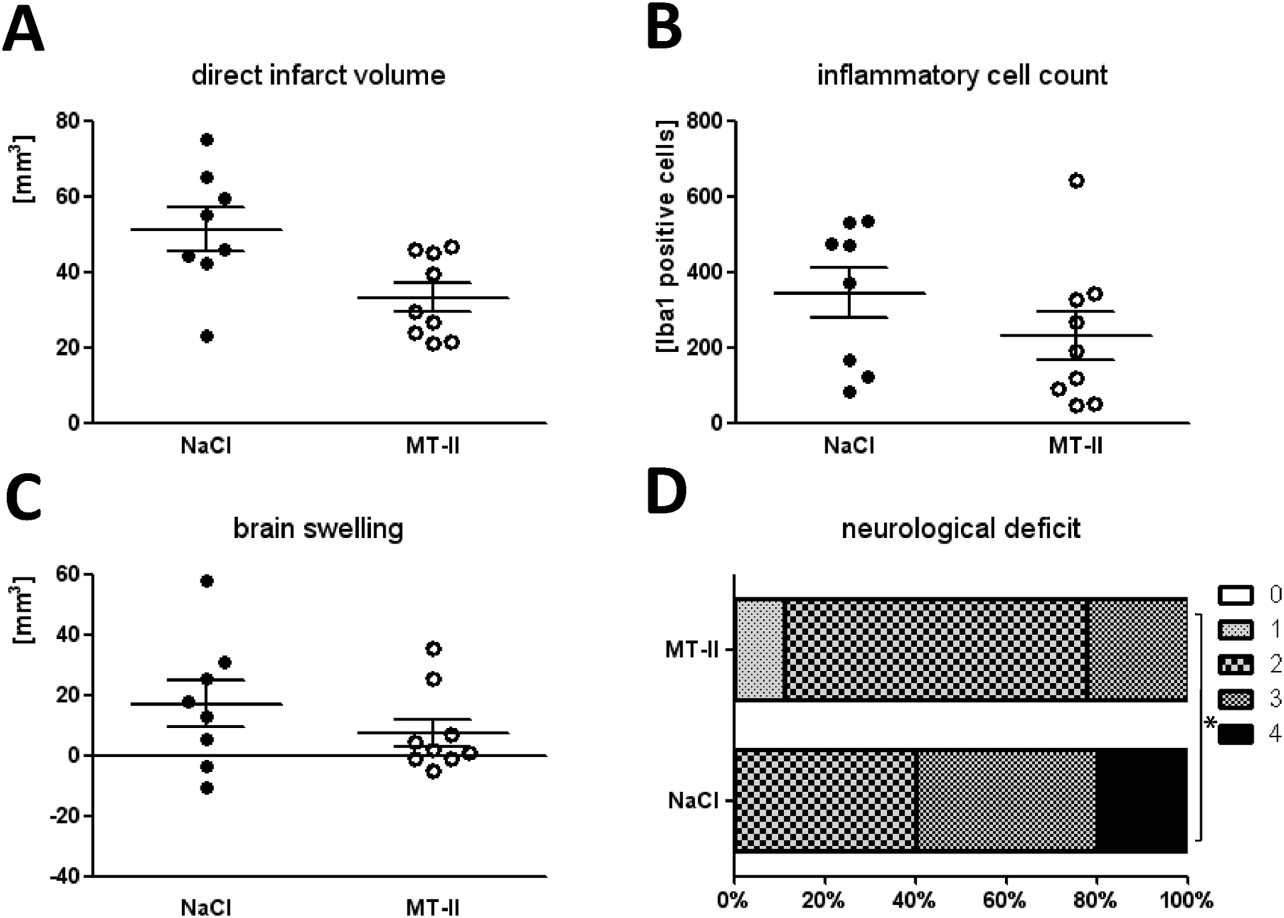
Effect of metallothionein-II *i*.*p*. treatment *in vivo* 72h after MCAO for 30 min. Infarct volumes of vehicle (0.9% NaCl) (n = 8) or MT-II *i*.*p*. (n = 9) treated male adult wildtype C57BL/6N mice after 60min MCAO and 48h reperfusion. **A**: Direct infarct volumes (*p-value < 0.05 as calculated by Mann Whitney U-Test, one-tailed). **B**: Inflammatory cell count in the ischemic hemisphere as determined by counting Iba1-positive cells in whole ischemic brain hemispheres of mice at 72h of reperfusion after 30min MCAO. Inflammatory cell accumulation (macrophages and activated microglia) was determined by counting Iba1-positive cells at interaural position No.III (distance to bregma 3.9mm) in the whole ischemic/ipsilateral hemisphere **C**: Brain swelling, as calculated by the difference between direct and indirect infarct volumes. Whereas indirect infarct volumes were calculated as the volume of the contralateral hemisphere minus the non-infarcted volume of the ipsilateral/ischemic hemisphere. (NaCl: vehicle-treated mice, MT-II: MT-II *i*.*p*.-treated wild-type mice). **D:** Neurological deficit of mice at 72h after induction of MCAO as determined by a modified Bederson score [41]: 0 represents no deficits, 1 represents an extension deficit in the contralateral leg, 2 a hemiparesis with circling, 3 loss of postural reflexes, and 4 death (*p < 0.05).

However, when mice were subjected to the mild stroke model (30 min MCAO and 72 h reperfusion), a significant reduction of both direct and indirect infarct volumes was observed in metallothionein-II treated mice (n = 9) when compared to vehicle-treated control mice (n = 8): direct infarct volume in the MT-II-treated group was 33 ± 4mm^3^, 51 ± 6mm^3^ in the vehicle-treated group (*p* = 0.02) (Fig 3A), and indirect infarct volume was 26 ± 3.5 mm^3^ (MT-II group), respectively 34 ± 3mm^3^(vehicle group) (*p* = 0.046) (data not shown). Interestingly, there was a tendency of reduced inflammatory cell accumulation in the ischemic hemisphere of MT-II *i*.*p*. treated mice at 72h after 30min MCAO when compared to the vehicle-treated mice, however without statistical significance (vehicle group: 344 ± 68; MT-II treated group: 230 ± 64; n_*vehicle*_ = 8; n_*MT-II*_ = 9; *p* = n.s.) (Fig 3B). Analysis of brain swelling in the mild MCAO model revealed a tendency towards reduced brain swelling in the MT-II treated group, without significance however (brain swelling in MT-II *i*.*p*. treated wild-type mice: 8 ± 4.5mm^3^, vehicle-treated group: 17 ± 8mm^3^; *p* = n.s.) (Fig 3C). Moreover, there was a significantly reduced neurological deficit in the metallothionein-treated group at 48h (*p* = 0.02) and 72h (*p* = 0.03) of reperfusion (n_*vehicle*_ = 10; n_*MT2*_ = 9) (Fig 3D).

### Evaluation of the effect of MT-II when applied i.v. and in combination with systemic thrombolysis in focal cerebral ischemia

Next, we wondered if a potential therapeutic use of metallothionein(MT)-II in stroke would interfere with systemic thrombolysis (rtPA *i*.*v*.), which is widely used in acute stroke patients [37]. Thus, we examined whether application of MT-II in combination with, or without, rtPA *i*.*v*. influences the outcome of experimental cerebral ischemia in mice. For that purpose, adult male C57BL/6N mice were subjected to 45min MCAO and treated *i*.*v*. within four different treatment groups at the start of reperfusion: either with vehicle only, with MT-II only, with rtPA only, or a with a combination of MT-II and rtPA *i*.*v*. Infarct volumes, neurological deficit and brain swelling were evaluated at 48h after MCAO.

**Fig 4:**
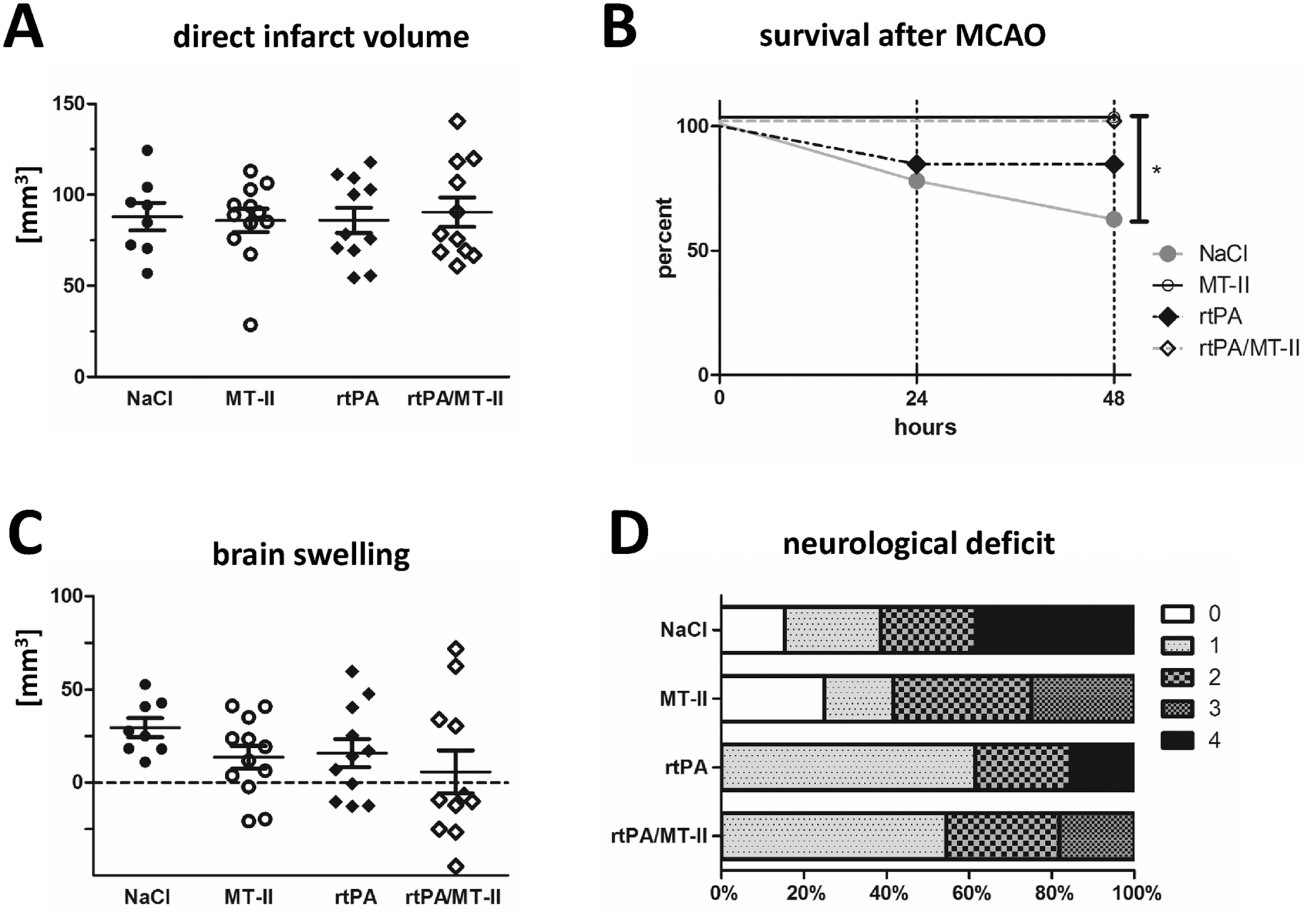
Effect of *i*.*v*. metallothionein-II treatment *in vivo* evaluated at 48h after MCAO for 45 min. Infarct volumes of vehicle (0.9% NaCl) (n = 8), MT-II (*i*.*v*.) (n = 12), rtPA (*i*.*v*.) (n = 11), or rtPA and MT-II co-application (*i*.*v*.) (n = 11) injected wildtype C57BL6/N male mice after 45min MCAO and 48h (**A**), Kaplan-Meier Analysis of animal survival (**B**), and brain swelling (**C**; as calculated by the difference of direct and indirect infarct volumes), and neurological deficit (**D**) in a standard model of cerebral ischemia (45min MCAO and 48h reperfusion) after intravenous treatment with metallothionein-II or vehicle only in combination with or without co-application of rtPA (*p = 0.0187; as calculated by log rank Mantel Cox test). Data **A** presented as scatter dot blots in combination with mean ± SEM. For comparison of infarct volumes and neurological deficit, ANOVA with Kruskal-Wallis test was used.

There was no alteration of direct infarct volumes in any of the four treatment groups (direct infarct volumes at 48h after MCAO: Vehicle-Group 88.0 ± 7.6mm^3^; MT-II-Group 86.0 ± 6.4mm^3^; rtPA-Group 86.0 ± 6.9mm^3^; MT-II/rtPA-Group 90.6 ± 8.1mm^3^; *p* = n.s.) (Fig 4A). Similar to the MT-II effects in the stringent MCAO model, *i*.*v*. application of MT-II led to only a tendency of decreased brain swelling (brain swelling: Vehicle 29.5 ± 5.1mm^3^; MT-II 13.5 ± 6.1mm^3^; rtPA 15.8 ± 7.6mm^3^; MT-II/rtPA 5.7 ± 11.5mm^3^; *p* = n.s.) (Fig 4C) and only a minor alteration of the neurological outcome (median of the Bederson score at 48h after MCAO: Vehicle 2; MT-II 2; rtPA 1; MT-II/rtPA 1; *p* = n.s.) (Fig 4D) in the MT-II-treated mice when compared to the groups without MT-II application, however without a statistical significance. Taken together, there was no obvious alteration of the effects of MT-II treatment when rtPA was co-administered, neither with regard to infarct volume, brain swelling, nor with regard to neurological deficit.

Interestingly however, there was an improvement of animal survival in the MT-II treated groups: whereas the effect in the MT-II/rtPA-*i*.*v*.*-*co-administration group did not reach significance when compared to rtPA only treated animals. MT-II*-i*.*v*. only treated animals had a significantly improved survival after MCAO when compared to the vehicle-only treated control group (*p* = 0.0187) (Fig 4B). Our *in vitro* experiments (Fig 1) and both *i*.*p*.-stroke models also revealed a similar improvement of cell, respectively animal survival. This observation argues for a general pro-survival role of metallothionein in stroke. Analysis of weight loss after MCAO or temperature does not reveal a difference in the 4 treatment groups (S2 Fig), which makes an infection-related effect of MT-II treatment unlikely, however it does not exclude that possibility.

**Fig 5:**
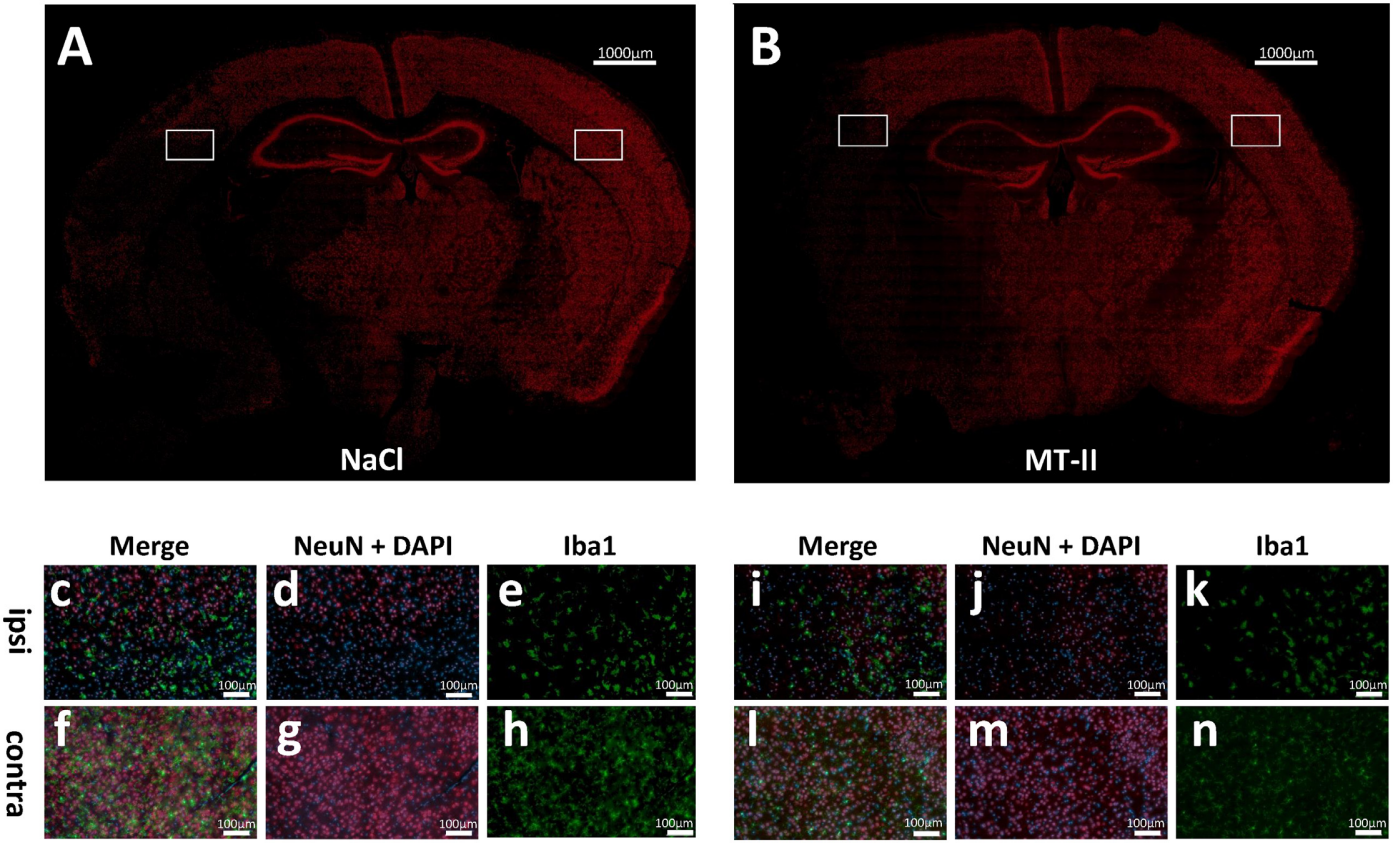
Evaluation by the use of immunohistofluorescence reveals no difference between MT-II and NaCl treated mice 48h after induction of MCAO. Neurons, as well as macrophages/microglia were stained by the use of anti-NeuN- (in red) and anti-Iba1-specific antibodies (in green) in brain slices derived at 48h reperfusion after induction of cerebral ischemia in adult male C57BL/6N wild-type mice, treated with NaCl only (**A**), or MT-II (5μg MT-II/10g body weight *i*.*v*. at start of reperfusion) (**B**). As shown in representative figures, neuron-specific staining (red) showed no significant alteration in ipsilateral (**c,d,i,j**) or contralateral/non-ischemic hemisphere after MT-II treatment when compared to NaCl-only treatment after MCAO (**f,g,l,m**). Moreover, Iba1-specific staining (for macrophages/microglia) does not reveal a clearly visible alteration after MT-II treatment (**k,n**) when compared to NaCl-only treatment (**e,h**) after MCAO.

Immunohistofluorescence also did not reveal a clear difference in NeuN-positive cell morphology or Iba1-positive cell morphology at 48h after MCAO, as shown in Fig 5. Analysis of inflammatory cell accumulation at 72h after induction of MCAO for 30min after vehicle or MT-II *i*.*p*. treatment also rather argues against a pure anti-inflammatory role of MT-II treatment in this model because inflammatory cell accumulation only correlates with infarct sizes, but not with regard to specific treatment (S3 Fig). A similar situation was found when inflammatory cell count (number of Iba1-positive cells in the ischemic hemisphere) was determined at 48h after 60min of MCAO (S4 Fig).

### Evaluation of the effects of MT-II treatment with regard to the expression of pro-inflammatory candidate genes by quantitative real-time PCR

**Fig 6:**
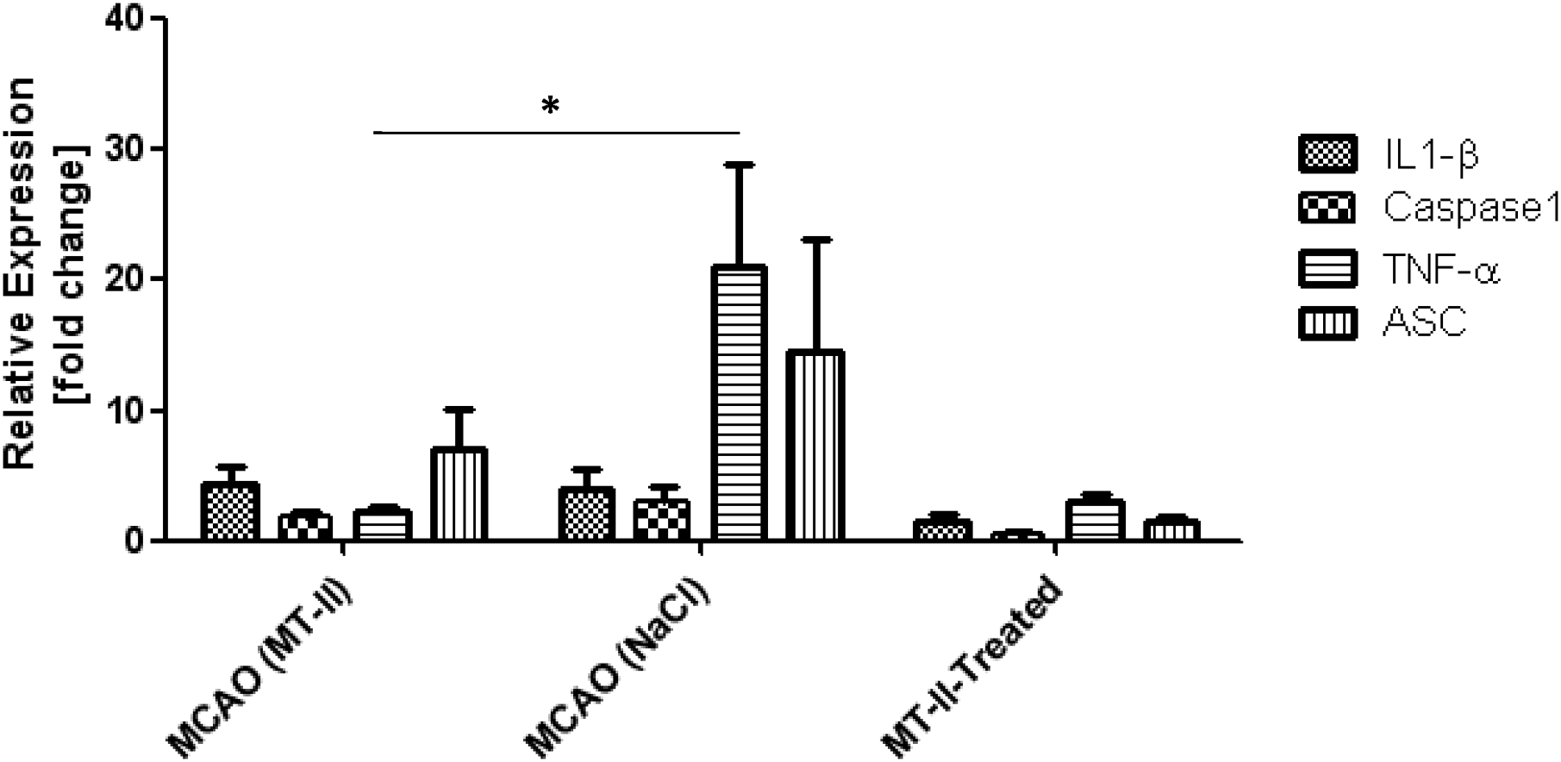
Gene expression analysis of pro-inflammatory proteins with real-time PCR. Relative mRNA expression of selected pro-inflammatory proteins was analysed in real-time PCR with gene-specific primers in whole mouse brain hemispheres of adult male wild-type C57BL/6N mice treated with either MT-II *i*.*p*. only without MCAO (MT-II-treated; n = 3), in mice at 72h after 30min MCAO that were treated with MT-II *i*.*p*. (n = 4), and in mice at 72h after 30min MCAO that were treated with vehicle only (n = 6). cDNA was separately isolated from each hemisphere. Relative Expression is presented as fold changes, calculated with the ΔΔCt-Method, normalized to HPRT mRNA expression and related to the non-ischemic/contralateral hemispheres as references. TNFα induction after MCAO is significantly reduced in mice that were treated with MT-II *i*.*p* when compared to vehicle-only treated mice (*p < 0.05, students one-side t-test). Data are shown as relative mRNA expression of each gene and is calculated from two technical replications of n = 3 to 6 mice for each condition (values are given as mean ± SEM).

Next, we wondered if gene expression analysis could give us a hint to which mechanism MT-II treatment protects against experimental stroke. For that purpose, mRNA expression of several pro-inflammatory candidate genes after 72h reperfusion and 30min MCAO were analyzed in ischemic hemispheres and compared to baseline expression in non-ischemic/contralateral hemispheres, normalized to the housekeeping gene HPRT–I (hypoxanthine phosphoribosyl transferase 1). Mice were treated with MT-II *i*.*p*. only, with MT-II *i*.*p*. after MCAO, or vehicle after MCAO (vehicle treated mice with MCAO: n_*vehicle+MCAO*_ = 6, body weight: 24g ± 2g; *i*.*p*. MT-II treated mice with MCAO: n_*MT-II + MCAO*_ = 4, body weight 24.8g ± 2g, i.p. MT-II treated mice [without MCAO]: n_*MT-IIonly*_ = 3, 23.5g ± 1g). Analysis of pro-interleukin(IL)-1β mRNA after MCAO did not reveal a significant alteration between MT-II treated and vehicle-only treated mice after stroke (relative IL-1β expression 72h after 30min MCAO: vehicle_*MCAO*_ 4 ± 1.5; MT-II_*MCAO*_: 4.4 ± 1.3; *p* = n.s.) (Fig 6). In contrast to IL-1β expression, real-time PCR with the use of caspase-1 specific primers revealed a tendency towards reduced caspase-1 mRNA expression after stroke in MT-II-treated mice when compared to vehicle only treated mice after stroke, however without statistical significance. Statistical significance was only observed when induction after MCAO was compared with baseline expression in healthy MT-II-only treated mice without MCAO (relative caspase1 mRNA expression 72h after 30min MCAO: vehicle_*MCAO*_ 3 ± 1.0; MT-II_*MCAO*_: 1.8 ± 0.4; *p* = n.s; MT-II *control* 0.5 ± 0.2) (Fig 6).

A similar situation was found when the mRNA expression of a further inflammasome compound was examined: mRNA expression of apoptosis-speck-like protein containing a CARD (ASC) seemed reduced in mice treated with MT-II i.p. after MCAO when compared with vehicle-only treated mice, however without statistical significance (relative ASC mRNA expression 72h after 30min MCAO: vehicle_*MCAO*_ 14.5 ± 8.5; MT-II_*MCAO*_: 7 ± 3; *p* = n.s.; MT-II *control* 1.5 ± 0.4) (Fig 6). TNF-α mRNA was also found to be induced after MCAO, but the degree of mRNA induction is significantly reduced in MT-II treated mice when compared to vehicle treated mice at 72h after 30min MCAO (relative TNF-α mRNA expression 72h after 30min MCAO: vehicle_*MCAO*_ 21 ± 8; MT-II_*MCAO*_: 2.0 ± 0.5; *p* = 0.03) (Fig 6).

## Discussion

Despite many efficient neuroprotective treatment strategies in cerebral ischemia, which have been proven in experimental stroke models, results of clinical studies were disillusioning [4]. This could be explained by the use of the wrong animal models, the wrong choice of target genes, ‘weak’ experimental data, or may also be explained by factors such as publication bias or other statistical problems [43].

MTs have been identified early in stroke research as proteins that are highly expressed and significantly induced in ischemic brain tissue. Both, experimental stroke models using metallothionein-over-expressing transgenic mice, as well as experiments with MT-I/II-deficient and MT-III-deficient mice, revealed a protective role of metallothionein in experimental stroke [19–21]. Proteins of the metallothionein family are highly conserved over many species, including mice, or drosophila. There are abundant reports of the protective abilities of metallothionein-I and–II in various diseases, including many of the central nervous system [5]. However, the exact mechanism of how metallothioneins act remains enigmatic despite speculations about a heavy metal scavenging function, modulation of immune responses, mechanisms which involve receptors such as the MT-receptor megalin, or a cytoprotective role of MT against nitric oxide (NO)-induced toxicity [14,44]. Moreover, many of the studies have been performed only *in vitro*.

There is only anecdotal data presently of *in vivo* use of exogenous metallothionein in stroke, despite strong protective effects in stroke models obtained by the use of transgenic mice [19–21]. Only recently, a small study was published during our work and reported on a protective effect of MT-II-application *i*.*p*. in a rat model of transient cerebral ischemia [27], but there is doubt if *i*.*p*. applied MT is able to reach the CNS due to its short half-life and its renal secretion [29]. MT-II rapidly enters the bloodstream after either *i*.*m*. or *i*.*p*. injection and recent studies have reported that *i*.*p*.-administered MT-I/II enters the central nervous system when the blood brain barrier is damaged by induction of EAE or traumatic brain injury, but is not able to cross the intact BBB [26,29]. Thus, interpretation of the significance of the therapeutic potential of MT in stroke up to date is hindered by the limited data, e.g. with regard to different species, stroke models, or ways of application.

Our results confirm the therapeutic potential of exogenous administered metallothionein in stroke models *in vitro* and *in vivo*. However, model-specific differences were observed and protective effect sizes did not always reach those observed with MT-I/II deficient or metallothionein-over-expressing transgenic mice [19,20], potentially explained by a more potent way of action if MT is over-expressed intracellularly instead of exogenously and systemically administered. Our experiments demonstrate that MT-II, when applied *i*.*p*. in adult wild-type mice, protects against ischemic stroke in a mild stroke model, whereas in a more severe stroke model, there was only a non-significant tendency towards protection.

Our data shows that pro-inflammatory TNF-α mRNA induction after MCAO is significantly reduced in mice treated with MT-II, a finding which is in agreement with the known immunmodulatory actions of MT-II [45]. Accordingly, MT-II was shown to suppress the ability of macrophages to stimulate T cell proliferation and was postulated to represent another form of danger signal [12]. Our finding that the neuroprotective effect of MT-II application is only significant in the model with 72h reperfusion and the suppressed expression of pro-inflammatory markers such as TNF-α, supports the idea that anti-inflammatory mechanisms substantially contribute to the neuroprotective effect of MT-II.

However, further experiments are required to evaluate if e.g. increasing the amount of the systemically applied MT-II or other routes of application could improve the observed neuroprotection after experimental stroke, before this promising protein could be tested in human stroke patients. Our data, also using intravenous application in combination with systemic thrombolysis (rtPA), did not reveal any detrimental effects. In contrast, MT-II treatment improved the survival rate in these studies. Our *in vitro* experiments, with primary neuronal cell cultures and both *i*.*p*.-stroke models, revealed a similar improvement of cell, respectively animal survival. This observation could argue for a general pro-survival role of MT in stroke and fits with the idea of MT being an end effector of cellular protection [28]. Highly interesting, Malavolta et al. [46] also found a significantly increased survival in very old mice that over-express MT, and Koumura et al. [21] observed a decline in the survival of MT-deficient mice after MCAO when compared to wild-type control mice, which argues for a more general mechanisms of the pro-survival effect of MT.

Unfortunately, the exact molecular mechanisms of the neuroprotective abilities of MT still remain obscure and the same is true for its systemic neuroprotective effects *in vivo*. This may relate to cadmium-detoxifying or zinc-regulating abilities [14], direct neuroprotective effects, but may also rely on the immunmodulatory effects of MT [12,28]. Nevertheless, we feel that despite nature being exceptionally reluctant to reveal the function of MT, as stated by Vallee [9], this should not hinder further research which is driven by the urgent need to improve the therapeutic options in stroke patients. MTs, which are present in a variety of species, which are highly expressed, which are rapidly induced under conditions of stress, and which own a very broad proven protective potential against various disorders or stressful conditions *in vitro and in vivo* could represent perfect candidates to deliver the first successful neuroprotective strategy in the clinic. However, further experimental studies are required before clinical stroke studies with application of MT could be initiated.

## Supporting Information

S1 Fig Indirect and direct infarct volumes in TTC-stained brain slices. (PDF)

S2 Fig Comparison of weight and temperature in different treatment groups after 45min MCAO. (PDF)

S3 Fig Correlation between inflammatory cell count and infarct sizes after 30min MCAO and 72h reperfusion with or without MT-II *i*.*p*. treatment. (PDF)

S4 Fig Correlation between inflammatory cell count and infarct sizes after 60min MCAO and 48h reperfusion with or without MT-II treatment. (PDF)

S1 Table Operative mortality of MCAO-treated mice. (PDF)
