# Supplementary material for: Assessment of the Therapeutic Potential of Metallothionein-II Application in Focal Cerebral Ischemia In Vitro and In Vivo
Source: PLoS One. 2015 Dec 14;10(12):e0144035. doi: 10.1371/journal.pone.0144035 (PMC4682799; doi:10.1371/journal.pone.0144035)
Supplement: S1 Fig — (PDF) [file pone.0144035.s001.pdf]

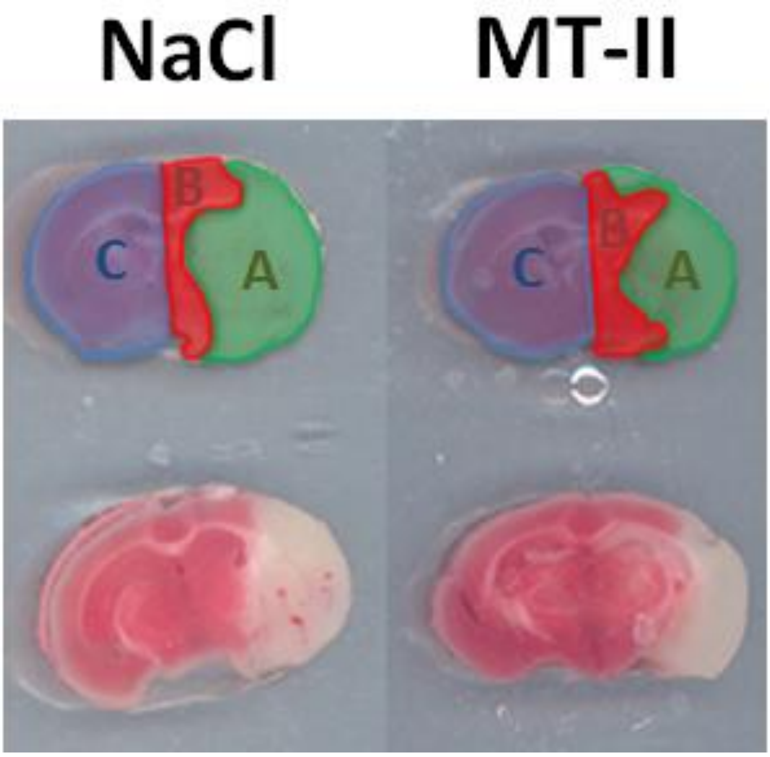

S1 Fig. Indirect and direct infarct volumes in TTC-stained brain slices. Representative slices (slice 2 and 3 out of 4) of NaCl and MT-II *i.v.* treated mice after 45min MCAO and 48h reperfusion. The size of area A represents the direct infarct. The indirect infarct is calculated by the difference of the size of the contralateral hemisphere (area C) minus the non-infarct area of the ipsilateral hemisphere (area B). Brain swelling is defined as the difference between direct and indirect infarct sizes.
