# Supplementary material for: Assessment of the Therapeutic Potential of Metallothionein-II Application in Focal Cerebral Ischemia In Vitro and In Vivo
Source: PLoS One. 2015 Dec 14;10(12):e0144035. doi: 10.1371/journal.pone.0144035 (PMC4682799; doi:10.1371/journal.pone.0144035)
Supplement: S2 Fig — (PDF) [file pone.0144035.s002.pdf]

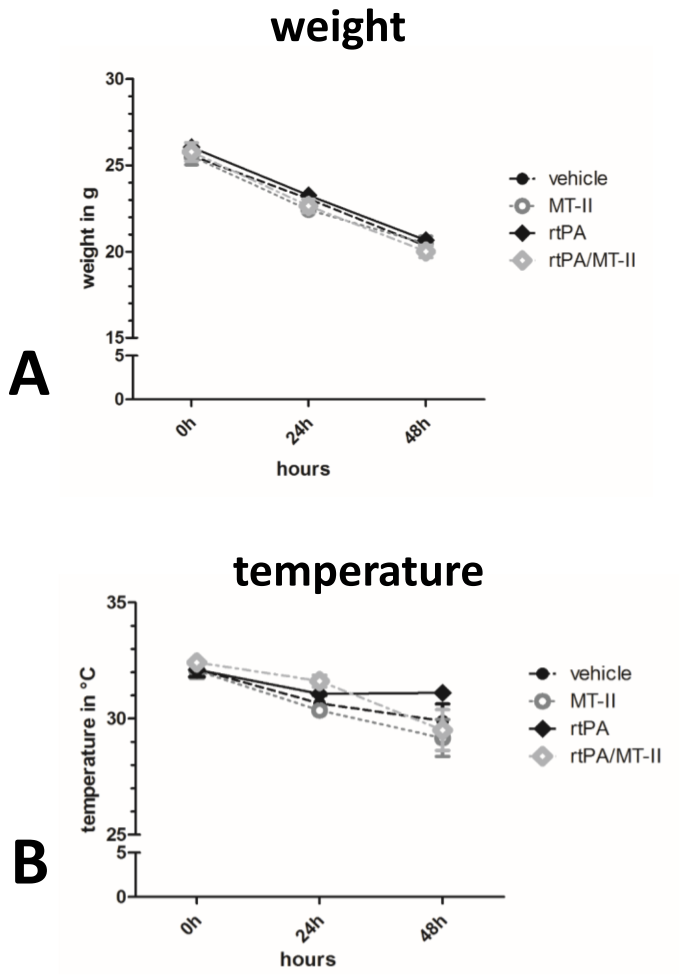

S2 Fig. Comparison of weight and temperature in different treatment groups after 45min MCAO. Adult male C57BL/6N wild-type mice were treated with either vehicle alone (NaCl), with metallothionein-II *i.v.* (MT), with rtPA *i.v.*, or with co-application of MT-II and rtPA *i.v.* (rtPA/MT-II). Data show the mean body weight (**A**) and mean temperature (**B**) in each group at different time points of reperfusion ($n_{vehicle}$ = 13; $n_{MT\text{-}II}$ = 12; n_{rtPA} = 13; $n_{rtPA/MT\text{-}II}$ = 11; values are given as mean ± SEM).
