# Supplementary material for: Assessment of the Therapeutic Potential of Metallothionein-II Application in Focal Cerebral Ischemia In Vitro and In Vivo
Source: PLoS One. 2015 Dec 14;10(12):e0144035. doi: 10.1371/journal.pone.0144035 (PMC4682799; doi:10.1371/journal.pone.0144035)
Supplement: S3 Fig — (PDF) [file pone.0144035.s003.pdf]

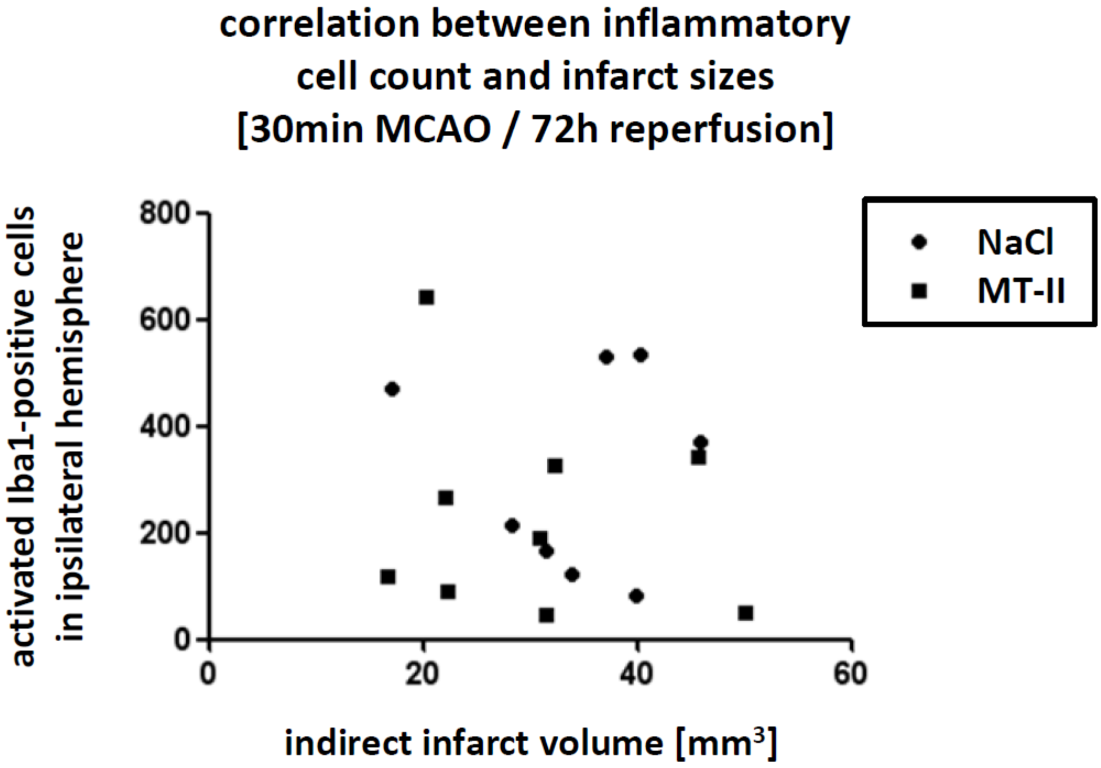

S3 Fig. Correlation between inflammatory cell count and infarct sizes after 30min MCAO and 72h reperfusion with or without MT-II *i.p.* treatment. Infarct sizes and number of Iba1-positive cells in the ischemic hemisphere were determined at 72h of reperfusion after induction of cerebral ischemia (MCAO) for 30min. Inflammatory cell count (macrophages and activated microglia) was determined as the number of Iba1-positive cells at interaural position No.III (distance to bregma 3.9mm) in the whole ischemic/ipsilateral hemisphere of mice 72h after induction of MCAO for 30min ($n_{vehicle}$ = 8; $n_{MT\text{-}II}$ = 9). (Spearman r_{NaCl} = 0.1429 and $r_{MT\text{-}II}$ = -0.1833)
