# Supplementary material for: Assessment of the Therapeutic Potential of Metallothionein-II Application in Focal Cerebral Ischemia In Vitro and In Vivo
Source: PLoS One. 2015 Dec 14;10(12):e0144035. doi: 10.1371/journal.pone.0144035 (PMC4682799; doi:10.1371/journal.pone.0144035)
Supplement: S4 Fig — (PDF) [file pone.0144035.s004.pdf]

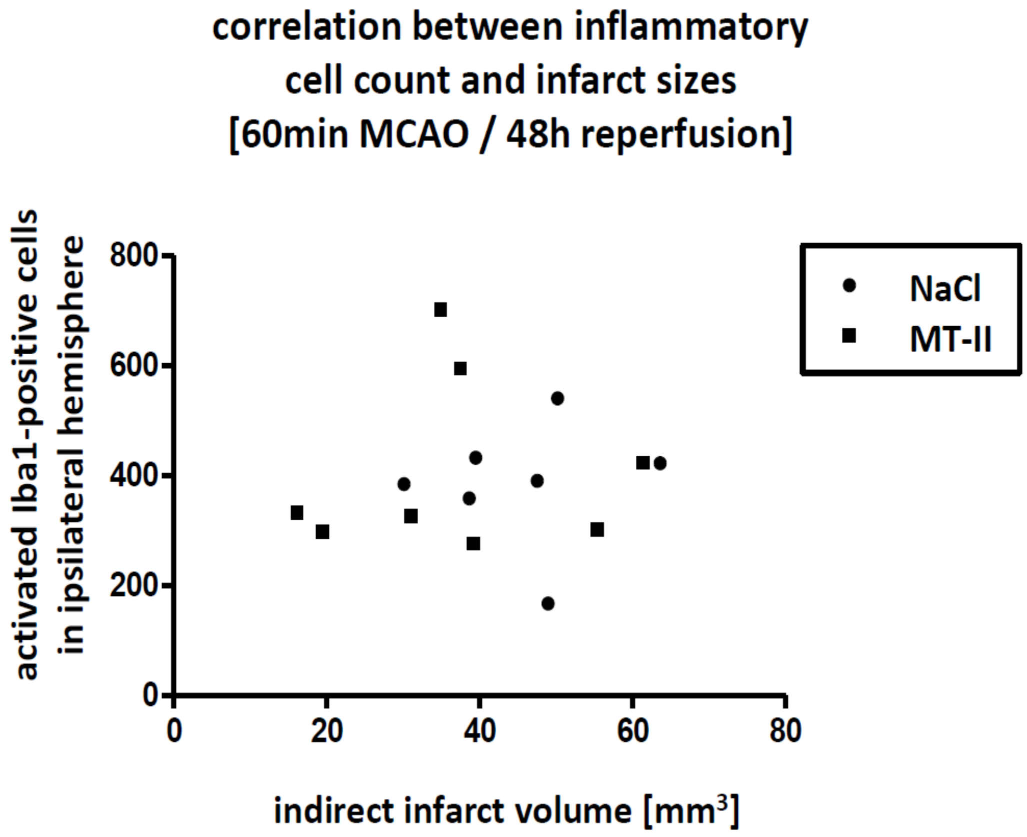

S4 Fig. Correlation between inflammatory cell count and infarct sizes after 60min MCAO and 48h reperfusion with or without MT-II treatment. Infarct sizes and number of Iba1-positive cells in the ischemic hemisphere were determined at 48h of reperfusion after induction of cerebral ischemia (MCAO) for 60min. Inflammatory cell count (macrophages and activated microglia) was determined as the number of Iba1-positive cells at interaural position No.III (distance to bregma 3.9mm) in the whole ischemic/ipsilateral hemisphere of mice 48h after induction of MCAO for 60min ($n_{vehicle} = 7$; $n_{MT\text{-}II} = 8$) (Spearman $r_{NaCl} = 0.3929$ and $r_{MT\text{-}II} = 0.02381$).
