# Supplementary material for: Assessment of the Therapeutic Potential of Metallothionein-II Application in Focal Cerebral Ischemia In Vitro and In Vivo
Source: PLoS One. 2015 Dec 14;10(12):e0144035. doi: 10.1371/journal.pone.0144035 (PMC4682799; doi:10.1371/journal.pone.0144035)
Supplement: S1 Table — (PDF) [file pone.0144035.s005.pdf]

**experiment**	**perioperative mortality**	**group**	**total number (n=)**	**postoperative mortality before end of reperfusion**
**60minMCAO, 48h Reperfusion**	4	NaCl i.p.	10	1
		MT-II i.p.	10	0
**30minMCAO, 72h Reperfusion**	1	NaCl i.p.	10	2
		MT-II i.p.	10	0
**45minMCAO, 48h Reperfusion**	11	NaCl i.v.	13	5
		MT-II i.v.	12	0
		rtPA i.v.	13	2
		rtPA/MT-II i.v.	11	0
**30minMCAO, 72h Reperfusion for qRT-PCR**	1	NaCl i.p.	6	0
		MT-II i.p.	6	1

S1 Table. Mortality of MCAO-treated mice. Perioperative mortality includes all mice that died until 6h of reperfusion. Postoperative mortality is defined as death between 6h and before end of the reperfusion
